# Interaction Patterns of Men Who Have Sex With Men on a Geosocial Networking Mobile App in Seven United States Metropolitan Areas: Observational Study

**DOI:** 10.2196/13766

**Published:** 2019-09-12

**Authors:** Nadia N Abuelezam, Yakir A Reshef, David Novak, Yonatan Hagai Grad, George R Seage III, Kenneth Mayer, Marc Lipsitch

**Affiliations:** 1 William F Connell School of Nursing Boston College Chestnut Hill, MA United States; 2 Department of Computer Science Harvard University Cambridge, MA United States; 3 DSN Consulting, LLC Boston, MA United States; 4 Department of Immunology and Infectious Disease Harvard TH Chan School of Public Health Boston, MA United States; 5 Division of Infectious Disease Brigham and Women's Hospital Harvard Medical School Boston, MA United States; 6 Department of Epidemiology Harvard TH School of Public Health Boston, MA United States; 7 The Fenway Institute Boston, MA United States; 8 Center for Communicable Disease Dynamics Boston, MA United States

**Keywords:** men who have sex with men, sexual behavior, race factors, population dynamics

## Abstract

**Background:**

The structure of the sexual networks and partnership characteristics of young black men who have sex with men (MSM) may be contributing to their high risk of contracting HIV in the United States. Assortative mixing, which refers to the tendency of individuals to have partners from one’s own group, has been proposed as a potential explanation for disparities.

**Objective:**

The objective of this study was to identify the age- and race-related search patterns of users of a diverse geosocial networking mobile app in seven metropolitan areas in the United States to understand the disparities in sexually transmitted infection and HIV risk in MSM communities.

**Methods:**

Data were collected on user behavior between November 2015 and May 2016. Data pertaining to behavior on the app were collected for men who had searched for partners with at least one search parameter narrowed from defaults or used the app to send at least one private chat message and used the app at least once during the study period. Newman assortativity coefficient (R) was calculated from the study data to understand assortativity patterns of men by race. Pearson correlation coefficient was used to assess assortativity patterns by age. Heat maps were used to visualize the relationship between searcher’s and candidate’s characteristics by age band, race, or age band and race.

**Results:**

From November 2015 through May 2016, there were 2,989,737 searches in all seven metropolitan areas among 122,417 searchers. Assortativity by age was important for looking at the profiles of candidates with correlation coefficients ranging from 0.284 (Birmingham) to 0.523 (San Francisco). Men tended to look at the profiles of candidates that matched their race in a highly assortative manner with R ranging from 0.310 (Birmingham) to 0.566 (Los Angeles). For the initiation of chats, race appeared to be slightly assortative for some groups with R ranging from 0.023 (Birmingham) to 0.305 (Los Angeles). Asian searchers were most assortative in initiating chats with Asian candidates in Boston, Los Angeles, New York, and San Francisco. In Birmingham and Tampa, searchers from all races tended to initiate chats with black candidates.

**Conclusions:**

Our results indicate that the age preferences of MSM are relatively consistent across cities, that is, younger MSM are more likely to be chatted with and have their profiles viewed compared with older MSM, but the patterns of racial mixing are more variable. Although some generalizations can be made regarding Web-based behaviors across all cities, city-specific usage patterns and trends should be analyzed to create targeted and localized interventions that may make the most difference in the lives of MSM in these areas.

## Introduction

### Background

Sexually transmitted infection (STI) and HIV transmission risk remain high among men who have sex with men (MSM) in the United States. MSM account for 68.2% of all primary and secondary syphilis cases in the United States in 2017 [1]. They account for 38.5% of isolates testing positive for *Neisseria gonorrhoeae* in the United States in 2017, up from 3.9% in 1989 [1]. Specific subpopulations at increased risk of infection include young black MSM and Latino MSM (MSM of color). Compared with their proportions in the US population, MSM of color have significantly higher burden of STIs than white MSM [1]. Rates of reported chlamydia cases were highest among black men (907.3/100,000), Native Hawaiian or Pacific Islander (376.6/100,000), and Asian men (402.6/100,000) in the United States when compared with white men (137.1/100,000) [1]. Gonorrhea rates are highest among black (660.7/100,000) and Asian (238.0/100,000) men in the United States [1].

Young black MSM are at highest risk for HIV and STI transmission in the United States despite reporting similar frequency of HIV risk behaviors as other groups [2,3]. Some believe that the structure of their sexual networks and partnership characteristics may be contributing to this high risk of HIV [4]. Assortative mixing (also known as homophily), which refers to the tendency of individuals to have partners from their own group, where a group can be defined by age, race, height, weight, or other characteristic factors involved in choosing a sexual partner, has been proposed as a potential explanation for disparities [5]. When assortative mixing by race is high, it can amplify STI and HIV prevalence differentials and may explain disparities in incidence among racial groups of MSM in the United States [4]. In a Web-based study of an ethnically diverse group of MSM, black MSM were more than 11 times as likely to have black partners than other racial group. In other studies of MSM in a number of US cities, black men were observed to have more black partners than any other racial group [6-9] and, in some cases, the majority of black men exclusively reported sex with black partners [10]. Nonblack MSM reported lesser preference for black partners in 1 study of MSM in San Francisco [11].

Assortative and disassortative mixing by age have also been found to be important to HIV transmission in particular contexts and are disparate by race [12], with more black MSM reporting sexual partners in a different age group (±5 years) than white men [13]. Black MSM in a number of studies were found to have a large number of partners that were in an older age group [9,10,13]. Evidence shows that having older sexual partners was associated with higher sexual risk behaviors in black MSM [12]. Odds of unprotected sex increased dramatically for young black MSM with partners who were 3 years older (a predictor only present for black MSM in this study) [4]. As black MSM have the highest HIV prevalence in the United States, this may potentially drive HIV transmission to young black MSM [12]. Studies to understand the relationship between sexual network composition and structure and risk for STIs are being conducted in these communities, but there remains a great deal of stigma associated with homosexuality and HIV that makes recruitment for studies difficult [14,15].

Understanding men’s preferences using Web-based apps may lead to better hypotheses and understanding of behaviors and risks associated with STI transmission in the real world. Increasingly, MSM use various forms of technology and the internet to locate sexual partners outside of physical local venues [16]. With the advancement of mobile technologies, geosocial networking (GSN) mobile apps have arisen that utilize global positioning system (GPS) to allow app users to find and chat with partners in their immediate city or community. Users post pictures and descriptions about themselves and are able to search for partners and chat with potential connections [17]. GSN apps allow men to *favorite* potential partners and identify when they may be in close proximity throughout their day [18]. Men’s use of these networks reflects the fact that they have control over who they interact with and respond to, and there is a relative ease with which they find partners [19]. For MSM of color, who have very few venues for socializing with other MSM because of stigma and taboo within their communities, Web-based platforms and apps play an important role in finding partners and providing a social atmosphere [20]. About one-third of all MSM have reported ever using a GSN app and 85% of those using apps engage with them daily [21,22].

Previous studies to estimate assortativity by age and race have focused on collecting egocentric and self-reported data from MSM through systemically or conveniently selected samples. Although important, these estimates are potentially limited by social desirability and recall biases of respondents [23-26], especially with regard to racial preferences. In addition, research has found that recruiting and retaining MSM of color in research using Web-based platforms has been difficult [26]. Studies that are able to observe behavior, without interfering with it, are ideal as they avoid the potential for bias and social desirability among the MSM being observed. Observing behavior on GSN apps provides a unique and novel perspective to collecting more robust objective data on sexual preferences and behavior.

### Objective

Our study was designed to objectively identify the age- and race-related search patterns of users of a diverse GSN mobile app in seven major metropolitan areas in the United States to better understand the disparities in STI and HIV risk in MSM communities.

## Methods

### Study Population

Data were collected on user behavior while using a diverse MSM GSN mobile app on an Apple or Android device between November 2015 and May 2016. Data entered by the user upon initiation of an account (including age, race, height, weight, and partner preferences) were collected where available. When signing up for a profile, individuals had the option of self-identifying their race as one of the following categories: Asian, Black, Caucasian, Latino, Middle Eastern, Mixed, Pacific Islander, or Other. We coded individuals into the following racial categories: Asian, black, white (indicated Caucasian), Latino, and other (indicated Middle Eastern, Mixed, Pacific Islander, or Other). Every time a user logged into the app and searched for a partner, information was collected on the parameters of the conducted search, including the following: GPS location of the searcher; the list of potential candidates resulting from the search; whether or not the user looked at the details of a candidate’s profile, favorited a candidate in the search list, or initiated a chat with a candidate in the search list; and the provided details of the candidate (including age, race, height, and weight). The data collected were composed of de-identified user identities for the searcher and the candidates resulting from the search.

Behavior on the app was collected for men who had searched for partners with at least 1 search parameter narrowed from defaults or used the app to send at least 1 private chat message to another individual and used the app at least once during the study period (November 2015 through May 2016). Users were categorized into 7 major metropolitan areas if their GPS coordinates fell within the metropolitan census tracts for the cities of Birmingham (Alabama), Boston (Massachusetts), Los Angeles (California), New York City (New York), San Francisco (California), Tampa (Florida), and Washington DC.

We had 2 main behavioral observations of interest: acquiring profile details and initiating a chat. If an individual clicked on a candidate’s name and looked at the profile details of a candidate in their search list, we considered this to be a situation in which details were acquired. If an individual initiated a chat with a candidate on their search list by sending a message to a candidate from the search list, we considered this to be a situation in which a chat was initiated (independent of chat length or duration).

### Human Subjects

An Institutional Review Board (Harvard TH Chan School of Public Health) and an independent ethics committee (Western Institutional Review Board) approved the study protocol and amendments for this study.

### Assortativity

For each city, a mixing matrix (*e*) was created by age and race, respectively: the *i* th to *j* th entry of *e* was the proportion of all searcher–candidate pairs for which the searcher fell in category *i* and the candidate fell in category *j*. The matrix *e* assessed the proportional cross-tabulation of age (or race) for searcher and candidate in each city, so that the sum of the entries in the matrix is 1. These mixing matrices were analyzed to better understand the patterns of activity on the GSN app by age and race.

A total of 2 quantities were calculated from the study data to understand assortativity patterns of men using the GSN app by age and race. Newman assortativity coefficient, R, a parameter used to assess the extent to which a population exhibits assortative, neutral, or disassortative sexual mixing patterns, was calculated from mixing matrices using the following equation:

R = Tr
*e* – ||
*e*^2^ || / 1 – ||
*e*^2^ ||

where R is the assortativity coefficient and *e* is the mixing matrix whose elements are *e*_ij_. Tr *e* is the trace of the mixing matrix (the sum of its diagonal elements), and || *e*^2^ || is the sum of the squared values of the elements in the mixing matrix.

An R value of 1 represents perfect assortativity in which people mix only with others having the same characteristics. An R value of 0 represents random mixing and an R value of −1 represents perfect disassortative mixing. We calculated Newman assortativity coefficient for racial categories and age categorized into 5-year increments up to the age of 40 years. As this measure compares mixing within the same group with mixing between groups, it is sensitive to the choice of band size for continuous measures such as age, with smaller age bands yielding lower values of assortativity. We, therefore, also calculated Pearson correlation coefficient to understand assortativity by age taken continuously to avoid this sensitivity and decrease it to outliers. Separate assortativity coefficients were calculated for each city of interest and each classification of the interaction (looking at candidate’s profile details or initiating a chat). When making reference to a specific group, such as men of Asian race/ethnicity, we define assortativeness for that group as a higher proportion of Asian candidates among the candidates of Asian searchers (the diagonal entry on the heat map) than among the candidates of all searchers (the corresponding marginal entry on the heat map).

On the basis of previous study, assortativity coefficients between 0.15 and 0.25 are considered minimally assortative, between 0.26 and 0.34 are considered moderately assortative, and 0.35 or larger are considered assortative [27,28].

Heat maps were used to help visualize the relationship between characteristics of the searcher and the candidate: age band, race, or age band and race. All values for heat maps were normalized by numbers of searchers in a category, thus the total value of a column (searcher) adds up to 100%. Besides (and above) each heat map, a column representing the distribution of candidates (and searchers) in the age, race, and age and race categories is shown. Heat maps were created for each city of interest and each classification of the interaction (looking at candidate’s profile details or initiating a chat).

## Results

### Descriptive Statistics

From November 2015 through May 2016 there were 2,989,737 searches in all seven major metropolitan areas among 122,417 searchers. The median number of searches per searcher in all cities was 3 to 4, but there were outliers in each city. In New York and Washington DC, for example, some searchers had over 15,000 searches in the study period. All searches resulted in 752,832 unique candidates. The age and race profiles of the searchers and candidates for each metropolitan area are outlined in Table 1. Notably, the majority of searchers in all metropolitan areas were aged between 21 and 29 years, with 5.59% (230/4114, Birmingham) to 9.38% (1014/10,929, San Francisco) of searchers in each city aged >40 years. The age distribution of searchers across metropolitan areas was relatively consistent.

The racial composition of the searchers varied by city. The majority of searchers self-identified as black or other, respectively, in Birmingham (77.72%, 3188/4102 and 13.99%, 574/4102) and Washington DC (64.96%. 17,699/27,245 and 20.67%, 5632/27,245) (Table 1). Boston had the largest proportion of white searchers (20.98%, 923/4400) and the second highest proportion of Asian searchers (22.52%, 991/4400) behind San Francisco (36.02%, 3902/10,833). Los Angeles had the highest proportion of searchers self-identifying as Latino (15.25%, 2003/13,132) and a high proportion self-identifying as other (22.96%, 3015/13,132) behind New York (24.98%, 10,490/41,991) and Tampa (23.44%, 1389/5925).

**Table 1:** Characteristics of men who have sex with men that searched, or were candidates themselves resulting from a search, for partners on a diverse social networking app focusing on such men in seven US metropolitan cities, using the app between November 2015 and May 2016.

Category			Birmingham	Boston	Los Angeles	New York	San Francisco	Tampa	Washington DC
**Instances (n)**			N=72,793	N=90,837	N=265,376	N=1,413,803	N=219,768	N=79,597	N=847,563
	Get details		14,363	12,842	40,583	183,085	30,488	18,901	132,581
	Chat		4978	3309	10,756	51,166	6759	6175	40,429
**Race, n (%)**									
	**Searchers**		N=4102	N=4400	N=13,132	N=41,991	N=10,833	N=5925	N=27,245
		White	211 (5.14)	923 (20.98)	1147 (8.73)	2978 (7.09)	1573 (14.52)	670 (11.31)	1867 (6.85)
		Black	3188 (77.72)	1088 (24.73)	4534 (34.53)	18,113 (43.14)	2022 (18.67)	3109 (52.47)	17,699 (64.96)
		Latino	35 (0.85)	473 (10.75)	2003 (15.25)	5751 (13.70)	919 (8.48)	533 (9.00)	934 (3.43)
		Asian	94 (2.29)	991 (22.52)	2433 (18.53)	4659 (11.10)	3902 (36.02)	224 (3.78)	1113 (4.09)
		Other	574 (13.99)	925 (21.02)	3015 (22.96)	10,490 (24.98)	2417 (22.31)	1389 (23.44)	5632 (20.67)
	**Candidates**		N=15,633	N=23,843	N=50,682	N=125,551	N=48,222	N=18,049	N=75,867
		White	555 (3.55)	3986 (16.72)	5974 (11.79)	13,239 (10.54)	7016 (14.55)	2069 (11.46)	6518 (8.59)
		Black	11,522 (73.70)	4517 (18.94)	13,641 (26.91)	46,512 (37.05)	7612 (15.79)	9455 (52.39)	41,175 (54.27)
		Latino	294 (1.88)	2048 (8.59)	6036 (11.91)	11,379 (9.06)	3467 (7.19)	1680 (9.31)	3671 (4.84)
		Asian	226 (1.45)	9648 (40.46)	15,275 (30.14)	29,621 (23.59)	22,333 (46.31)	673 (3.73)	9168 (12.08)
		Other	3036 (19.42)	3644 (15.28)	9756 (19.25)	24,800 (19.75)	7794 (16.16)	4172 (23.11)	15,335 (20.21)
**Age (years), n (%)**									
	**Searchers**		N=4114	N=4408	N=13,183	N=42,305	N=10,929	N=5937	N=27,546
		18-20	646 (15.70)	553 (12.55)	1119 (8.49)	4371 (10.33)	1235 (11.30)	815 (13.73)	2641 (9.59)
		21-24	1152 (28.00)	1205 (27.34)	3344 (25.37)	10,375 (24.52)	2627 (24.04)	1586 (26.71)	6017 (21.84)
		25-29	1215 (29.53)	1364 (30.94)	4686 (35.55)	14,700 (34.75)	3291 (30.11)	1906 (32.10)	9219 (33.47)
		30-34	597 (14.51)	607 (13.77)	2077 (15.76)	6617 (15.64)	1812 (16.58)	760 (12.80)	4725 (17.15)
		35-39	274 (6.66)	315 (7.15)	977 (7.41)	3359 (7.94)	950 (8.69)	402 (6.77)	2469 (8.96)
		40 and older	230 (5.59)	364 (8.26)	980 (7.43)	2883 (6.81)	1014 (9.38)	468 (7.88)	2475 (8.98)
	**Candidates**		N=15,814	N=24,114	N=51,124	N=126,942	N=48,770	N=18,151	N=76,908
		18-20	2094 (13.24)	2861 (11.86)	4574 (8.95)	12197 (9.61)	4278 (8.77)	2264 (12.47)	7467 (9.71))
		21-24	4704 (29.75)	6783 (28.13)	12,593 (24.63)	30,259 (23.84)	11,421 (23.42)	4940 (27.22)	17,987 (23.39)
		25-29	5134 (32.46)	7959 (33.01)	17,749 (34.72)	43,800 (34.50)	16,202 (33.22)	6047 (33.31)	26,530 (34.50)
		30-34	2173 (13.74)	3421 (14.19)	8223 (16.08)	20,725 (16.33)	8483 (17.39)	2463 (13.57)	12,644 (16.44)
		35-39	915 (5.79)	1658 (6.88)	4247 (8.31)	10,548 (8.31)	4441 (9.11)	1223 (6.38)	6416 (8.34)
		40 and older	794 (5.02)	1432 (5.38)	3738 (7.31)	9413 (7.42)	3945 (8.09)	1214 (6.69)	5864 (7.62)

### Statistics for Age

Assortativity by age, as measured by Pearson correlation coefficient, ranged from 0.117 (Tampa) to 0.210 (Los Angeles) across all search activities.

#### Details

**Figure 1:**
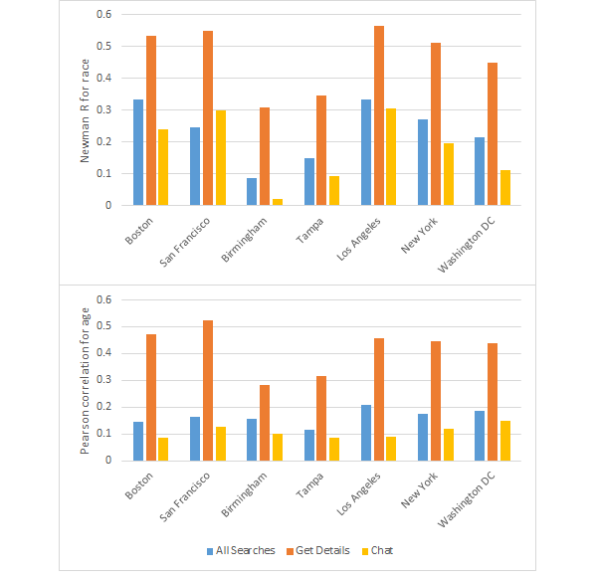
Assortativity results for race (top) and age (bottom) for each of the seven metropolitan areas.

**Figure 2:**
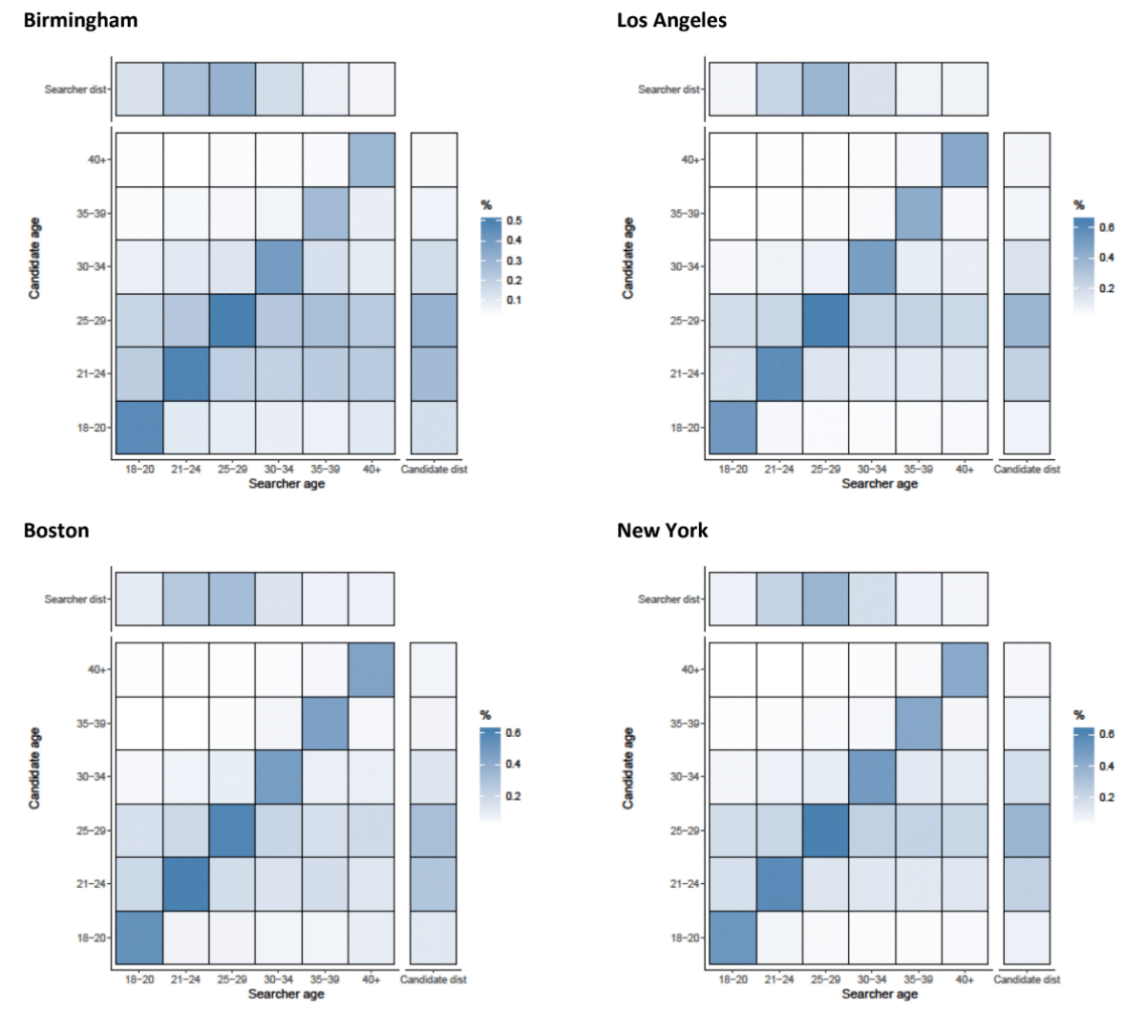
Assortativity by age (years) for details that are acquired by searchers on candidates in Birmingham, Los Angeles, Boston, and New York. Heat maps show the relationship between searcher’s age band and the candidate’s age band. All values are normalized by numbers of searchers in each age category (the total value of a column adds up to 100%). Besides each heat map, a column representing the distribution of candidates in the age band and above each heat map, a row representing the distribution of searchers is shown.

**Figure 3:**
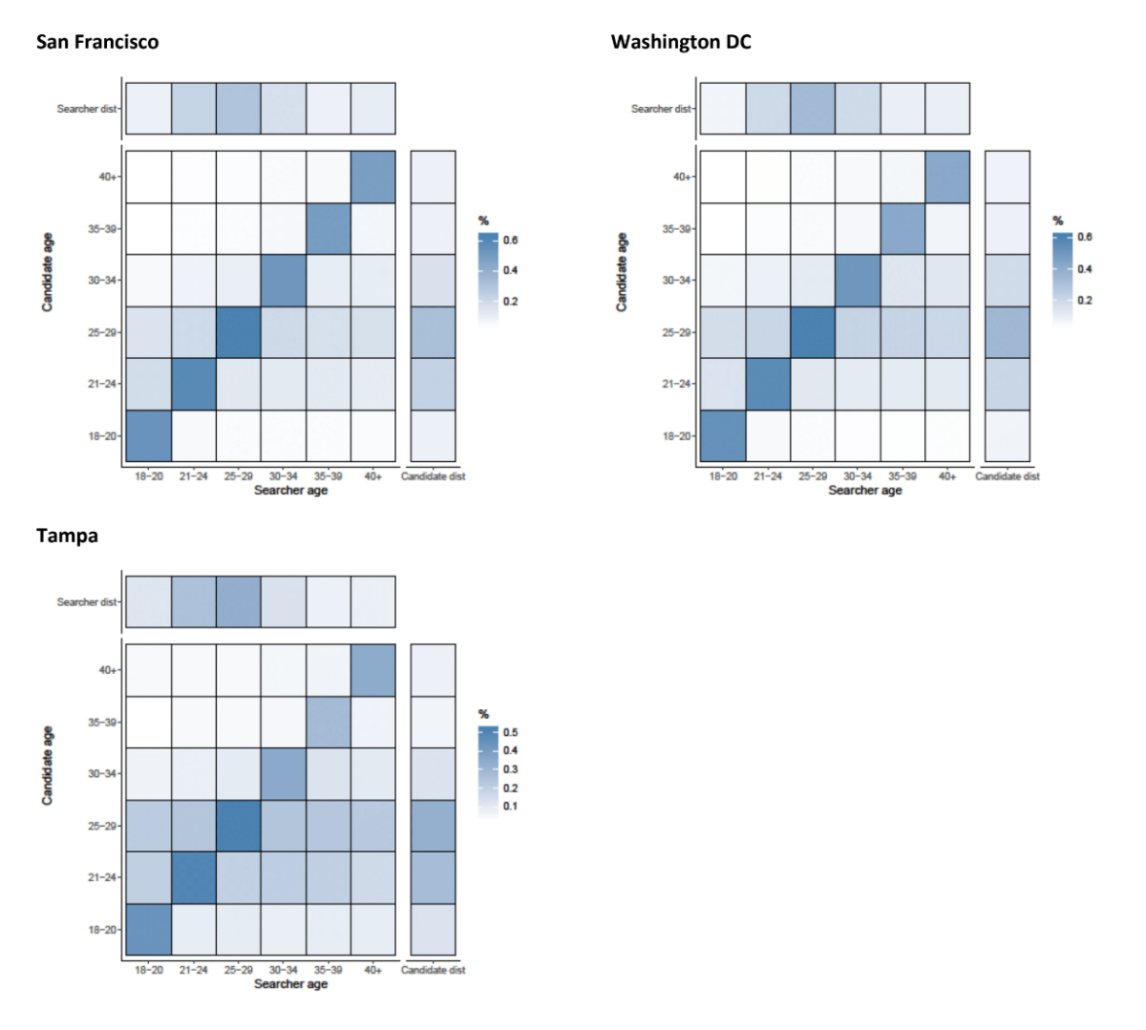
Assortativity by age (years) for details that are acquired by searchers on candidates in San Francisco, Washington DC, and Tampa. Heat maps show the relationship between searcher’s age band and the candidate’s age band. All values are normalized by numbers of searchers in each age category (the total value of a column adds up to 100%). Besides each heat map, a column representing the distribution of candidates in the age band and above each heat map, a row representing the distribution of searchers is shown.

Assortativity by age was important for looking at the profile details of candidates, with correlation coefficients ranging from 0.284 (Birmingham) to 0.523 (San Francisco) (Figure 1). Across all cities, searchers looked at the profiles of candidates that were from their own age category (suggesting assortative preference) and men aged between 21 and 29 years in all areas (seen by the horizontal striping across the heat maps for these 2 age groups) (Figures 2 and 3). Mean absolute age differences between the searcher and the candidate ranged from 4.33 (Boston, SD 7.2) to 5.65 (Tampa, SD 8.1) when details were examined (results not shown).

#### Chats

**Figure 4:**
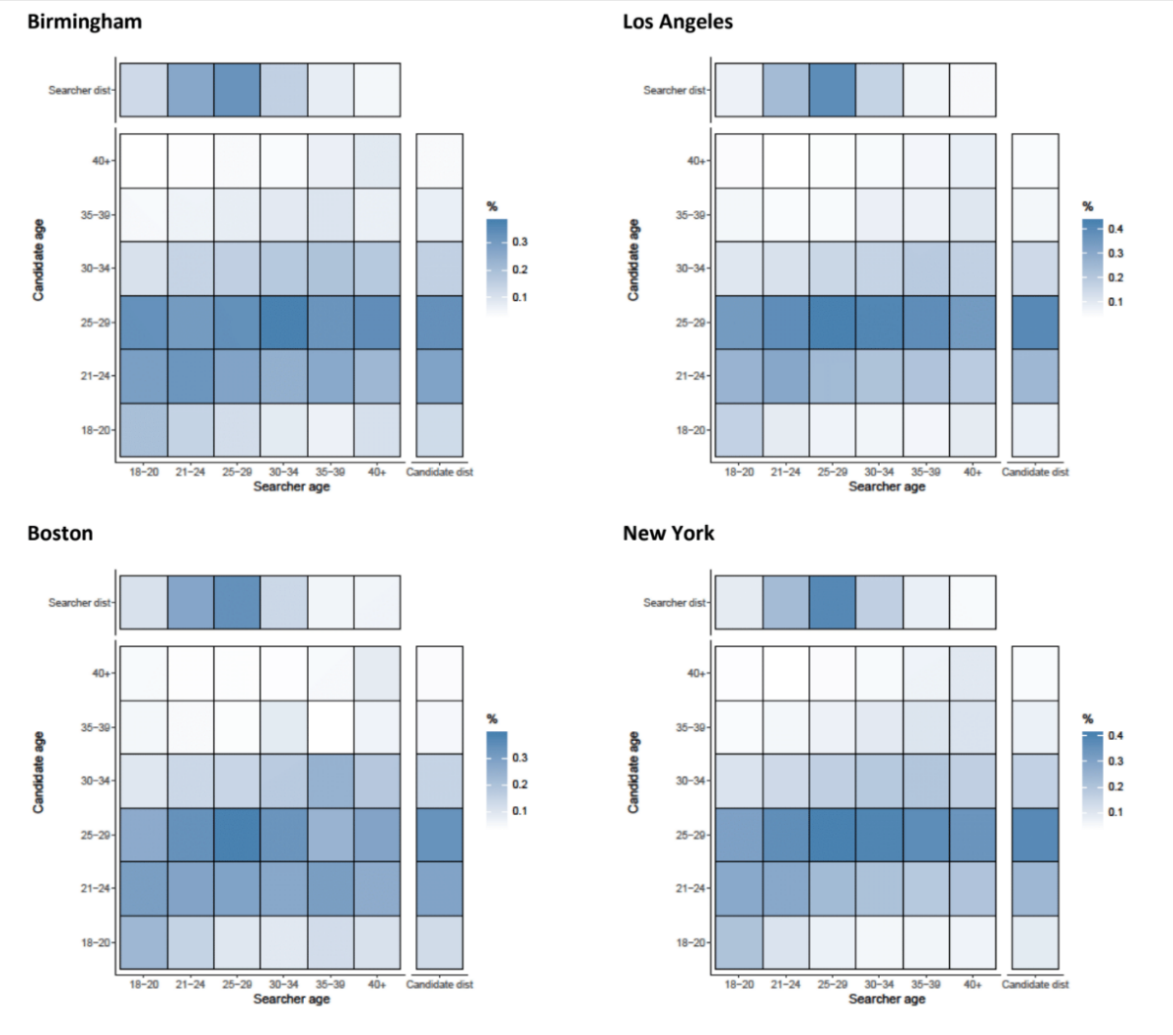
Assortativity by age (years) for chats initiated by searchers with candidates in Birmingham, Los Angeles, Boston, and New York. Heat maps show the relationship between searcher’s age band and the candidate’s age band. All values are normalized by numbers of searchers in each age category (the total value of a column adds up to 100%). Besides each heat map, a column representing the distribution of candidates in the age band and above each heat map, a row representing the distribution of searchers is shown.

**Figure 5:**
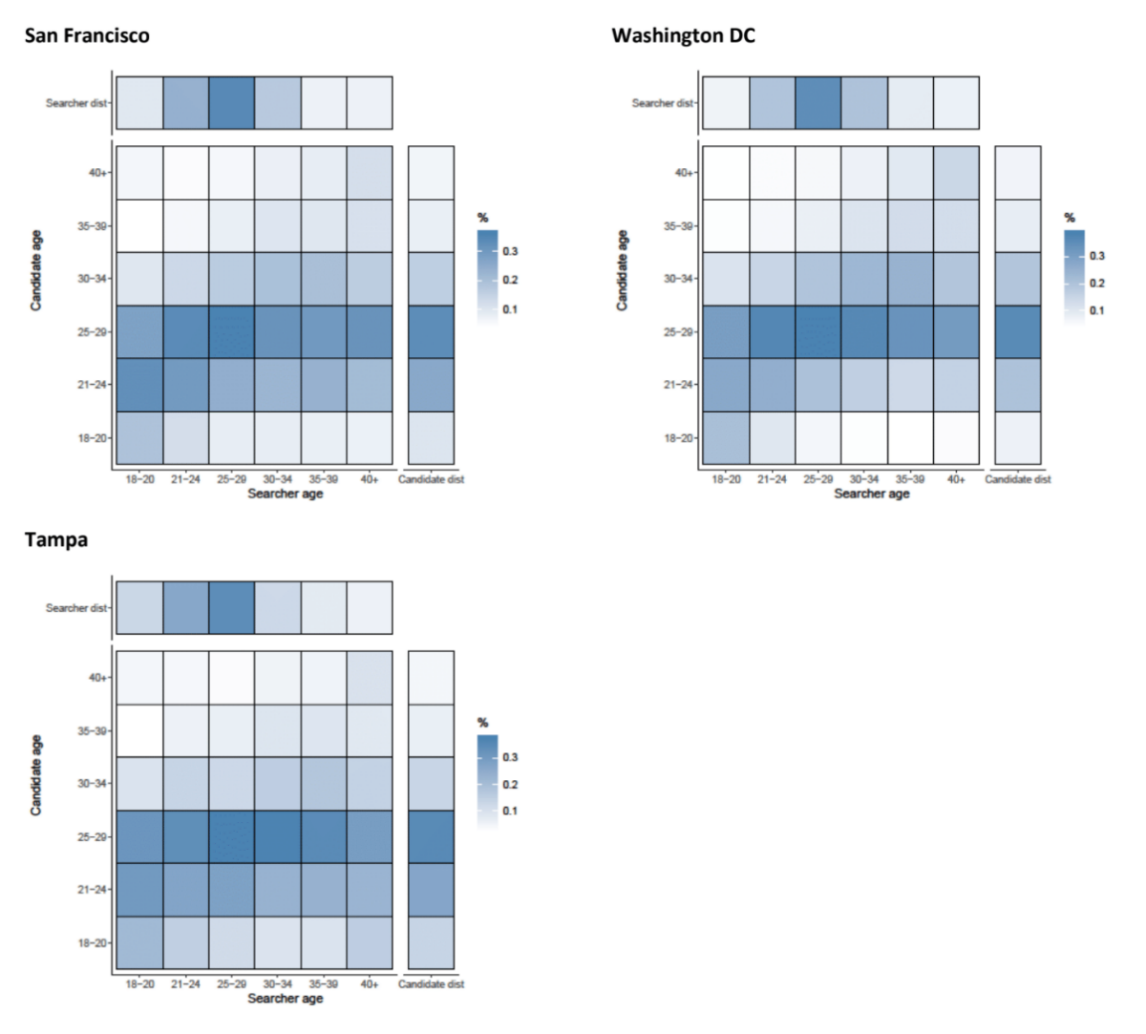
Assortativity by age (years) for chats initiated by searchers with candidates in San Francisco, Washington DC, and Tampa. Heat maps show the relationship between searcher’s age band and the candidate’s age band. All values are normalized by numbers of searchers in each age category (the total value of a column adds up to 100%). Besides each heat map, a column representing the distribution of candidates in the age band and above each heat map, a row representing the distribution of searchers is shown.

When initiating chats with candidates, searchers were not highly selective by age, with correlation coefficients ranging from 0.085 (Boston) to 0.148 (Washington DC) (Figure 1). Younger men were more often chosen for chats in all cities, with 25 to 29-year-olds being more often chosen on chats than older men. Searchers from all age categories initiated chats with young men (aged 21-29 years), as can be seen by the horizontal stripes in the chat initiated heat maps (Figures 4 and 5). There was some age assortativity for men aged 25 to 29 years initiating chats with other men aged 25 to 29 years, as can be seen by the darker shaded box on the diagonal for these age groups in most cities, compared with the box for this age at the right margin, which gives the frequency with which this age group was chatted with by searchers of all ages. Chats were initiated with men from younger age categories most commonly in Boston (36.78%, 1217/3309) and least commonly in New York (34.72%, 17,763/51,166; results not shown). Chats were initiated with men from older age categories most commonly in San Francisco (34.39%, 2325/6759) and least commonly in Los Angeles (31.47%, 3385/10,756; results not shown). Mean absolute age differences between searcher and candidates ranged from 6.49 (New York, SD 7.9) and 7.09 (San Francisco, SD 8.1) when chats were initiated (results not shown).

### Newman Assortativity for Race

Across all searches in all of the metropolitan areas examined, there was evidence for moderate assortativity by race with Newman assortativity coefficient R>0 for all cities. Across all search activities, R was highest in Los Angeles (0.33) and Boston (0.335) and lowest in Birmingham (0.088) and Tampa (0.149) (Figure 1).

#### Details

**Figure 6:**
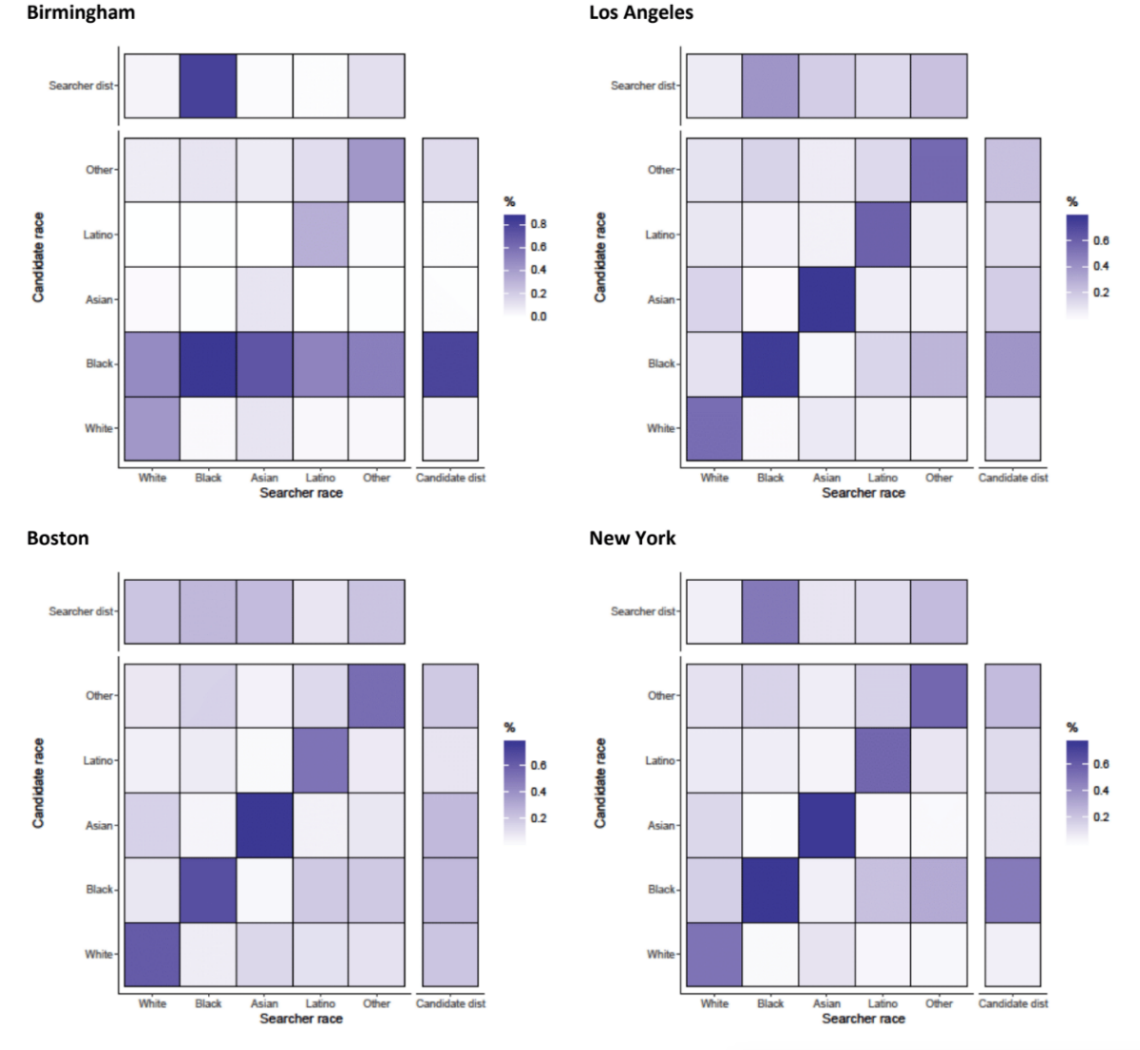
Assortativity by race for details that are acquired by searchers on candidates in Birmingham, Los Angeles, Boston, and New York. Heat maps show the relationship between searcher’s race group and the candidate’s race group. All values are normalized by numbers of searchers in each race category (the total value of a column adds up to 100%). Besides each heat map, a column representing the distribution of candidates in the race group and above each heat map, a row representing the distribution of searchers is shown.

**Figure 7:**
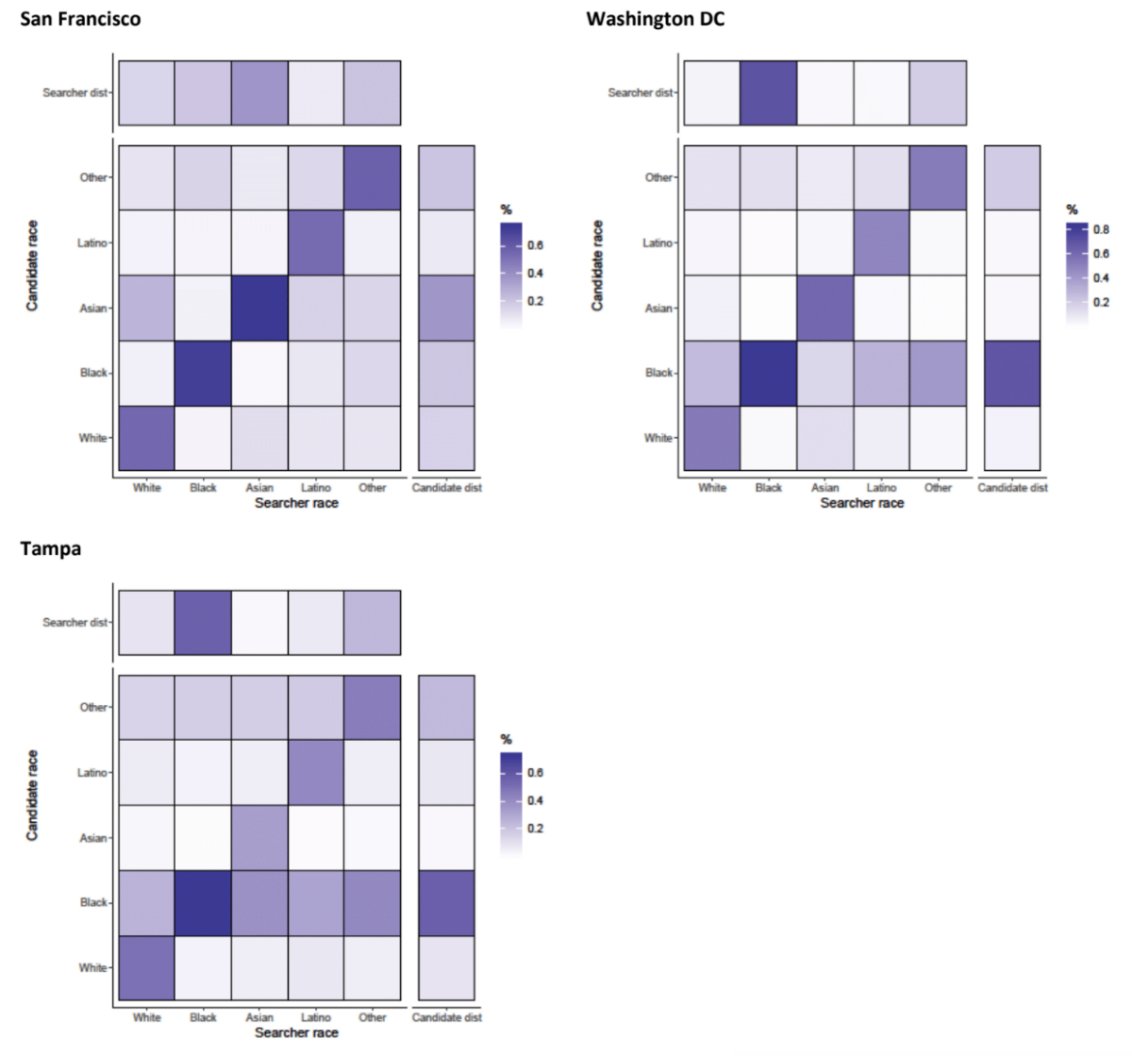
Assortativity by race for details that are acquired by searchers on candidates in San Francisco, Washington DC, and Tampa. Heat maps show the relationship between searcher’s race group and the candidate’s race group. All values are normalized by numbers of searchers in each race category (the total value of a column adds up to 100%). Besides each heat map, a column representing the distribution of candidates in the race group and above each heat map, a row representing the distribution of searchers is shown.

Men tended to look at the details of candidates that matched their race in a highly assortative manner with Newman R coefficients ranging from 0.310 (Birmingham) to 0.566 (Los Angeles) (Figure 1). In Birmingham and Tampa, all searchers (regardless of race) viewed black candidates’ profiles, potentially a byproduct of the proportion of candidates who were black in these cities (Figures 6 and 7). The majority of candidates in Birmingham, New York, Tampa, and Washington DC were black, whereas the majority of searchers in San Francisco were Asian (Figure 4). Black searchers looked at details for black candidates most commonly in Washington DC (83.20%, 110,307/132,581) and Birmingham (85.60%, 12,295/14,363) and least commonly in Boston (66.70%, 8566/12,842; results not shown).

#### Chats

**Figure 8:**
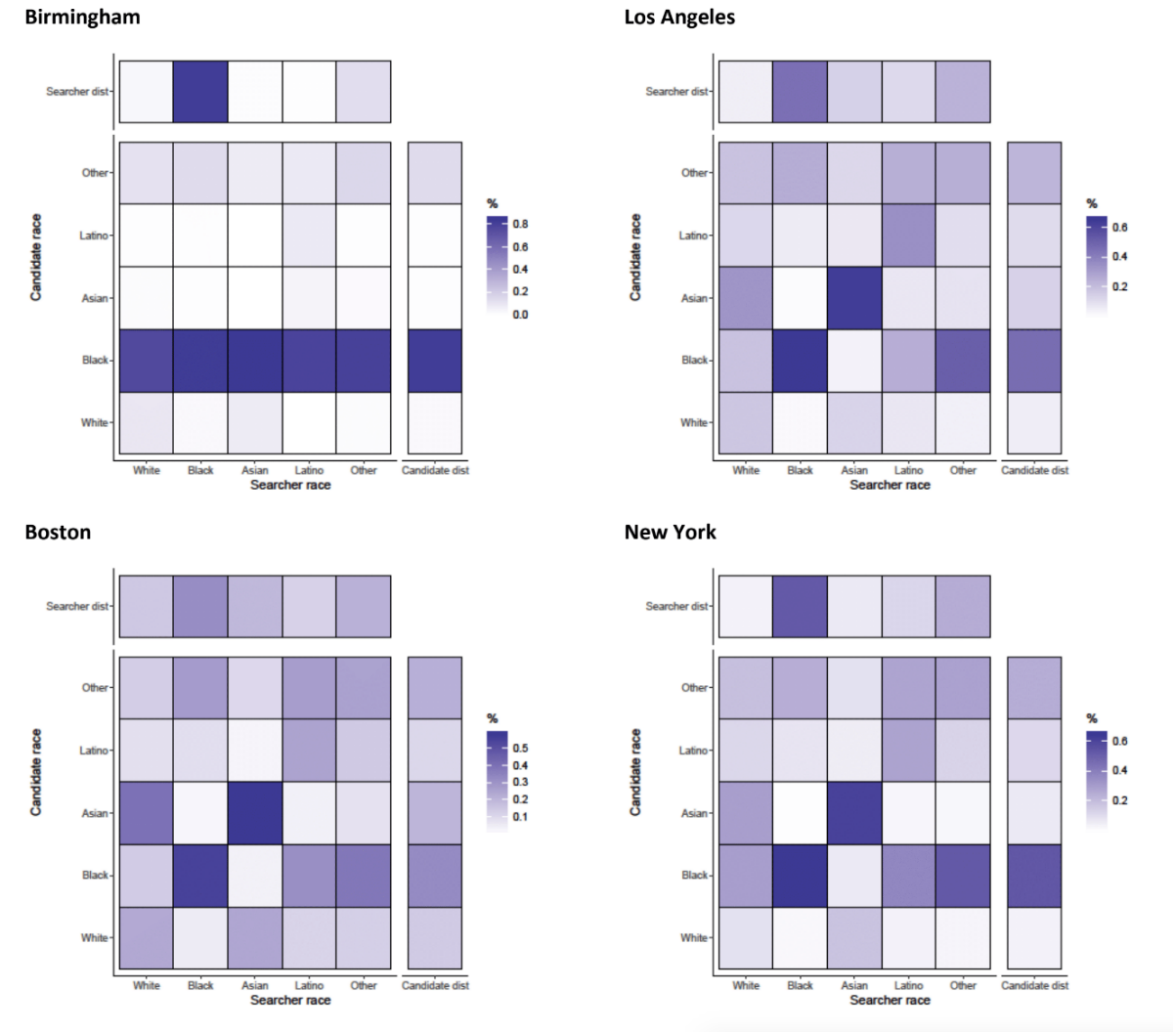
Assortativity by race for chats that are initiated by searchers on candidates in Birmingham, Los Angeles, Boston, and New York. Heat maps show the relationship between searcher’s race group and the candidate’s race group. All values are normalized by numbers of searchers in each race category (the total value of a column adds up to 100%). Besides each heat map, a column representing the distribution of candidates in the race group and above each heat map, a row representing the distribution of searchers is shown.

**Figure 9:**
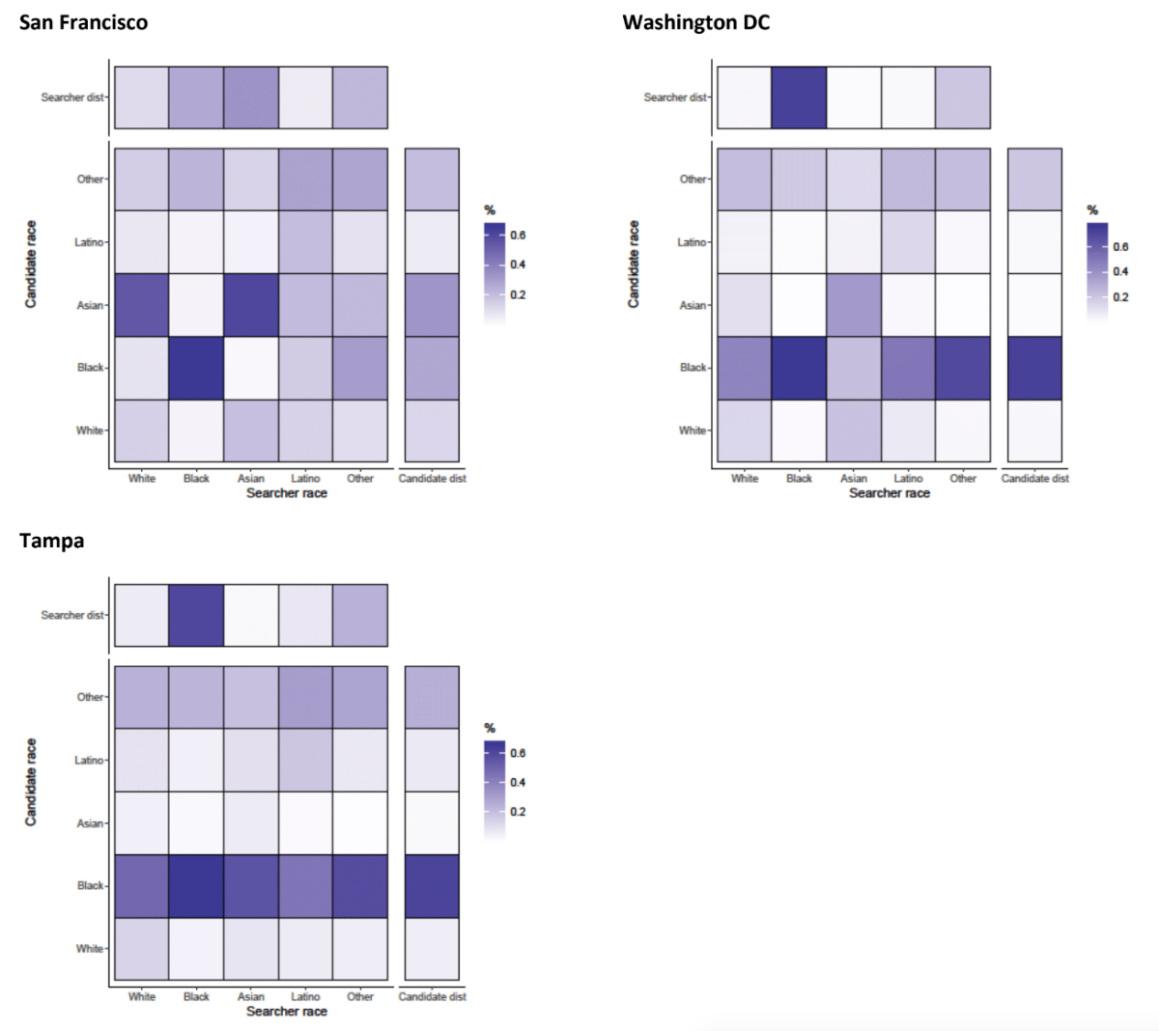
Assortativity by race for chats that are initiated by searchers on candidates in San Francisco, Washington DC, and Tampa. Heat maps show the relationship between searcher’s race group and the candidate’s race group. All values are normalized by numbers of searchers in each race category (the total value of a column adds up to 100%). Besides each heat map, a column representing the distribution of candidates in the race group and above each heat map, a row representing the distribution of searchers is shown.

For the initiation of chats, race appeared to be assortative for some groups with R ranging from 0.023 (Birmingham) to 0.305 (Los Angeles) (Figure 1). Asian searchers were most assortative in initiating chats with Asian candidates in Boston, Los Angeles, New York, and San Francisco (Figures 8 and 9). In Birmingham and Tampa, searchers from all races tended to initiate chats with black candidates, as evidenced by the dark horizontal line in the heat maps for black candidate race. This reduced overall assortativity for chats in these cities. All cities showed strong assortativity among black searchers and candidates, with black searchers chatting with black candidates most commonly in Birmingham (82.50%, 4107/4978) and least commonly in Boston (54.82%, 1814/3309). Searchers with an “other” self-classified race tended to chat with black candidates in Los Angeles, New York, and Washington DC white searchers initiated chats with Asian candidates in Boston and San Francisco but not in other cities. There was slight evidence that Latino searchers were assortative in initiating chats with other Latinos in Boston, Los Angeles, and New York.

### Race and Age

**Figure 10:**
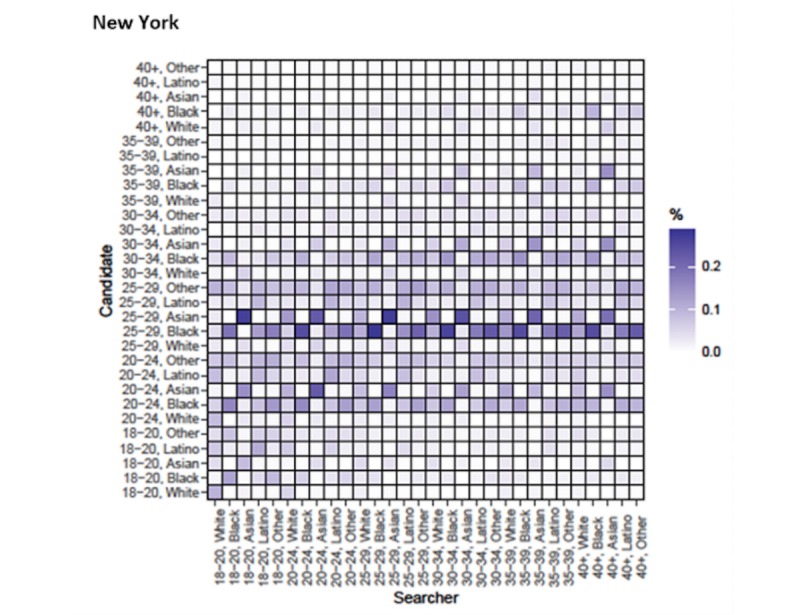
Assortativity by age (years) and race for chats initiated in Washington DC. Heat maps show the relationship between searcher’s age and race group and the candidate’s age and race group. All values are normalized by numbers of searchers in each age and race category (the total value of a column adds up to 100).

**Figure 11:**
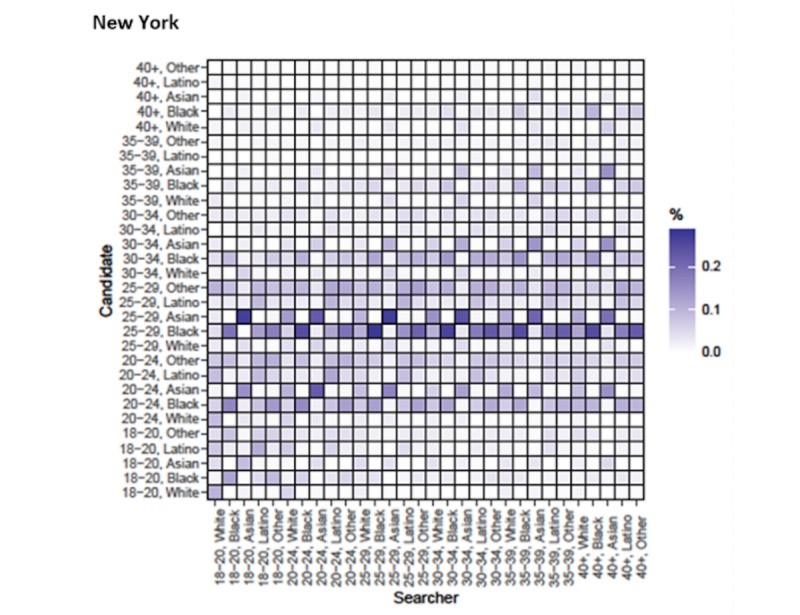
Assortativity by age (years) and race for chats initiated in New York. Heat maps show the relationship between searcher’s age and race group and the candidate’s age and race group. All values are normalized by numbers of searchers in each age and race category (the total value of a column adds up to 100).

When examining heat plots of both age and race together, we see that across searchers of various age/race combinations, 25 to 29-year-old black men are the most highly chatted with candidates, with similar trends for black men aged 20 to 24 and 30 to 34 years (Figures 10 and 11). In New York, black and Asian searchers are highly assortative by age when initiating chats but also more likely to chat with 25 to 29-year-old black and Asian candidates, respectively (Figure 11). Washington DC was the only city to show evidence of older black candidates (aged >35 years) being actively chatted with across all age and race groups of the searchers.

## Discussion

### Principal Findings

Our results indicate that the age preferences of MSM are relatively consistent across cities, that is, younger MSM profiles are more likely to be viewed and chatted with compared with older MSM, but the patterns of racial mixing are more variable. Specifically, we see that men tend to look at profiles and access details for other men on the platform from similar age, race, and age/race subgroups, but men initiate chats with men aged between 20 and 29 years most often, independent of searcher age. Assortativity patterns with regard to age were similar to other studies done with MSM across the country. A study by Tieu et al [10] in 6 US cities found that assortativity for age was estimated to be 0.20, within the range of age assortativity coefficients calculated in our study. We also see that Asian and black men tend to initiate chats with other Asian and black men in most cities. Cities such as Birmingham and Tampa may have different racial mixing patterns, with young black candidates being chatted with more often than other subgroups of the population (independent of searcher race).

Although we cannot, with certainty, generalize the interaction patterns of men in Web-based settings to real-world settings, Web-based partnership selection behavior may inform our understanding of real-world patterns in STI spread. Our observation of behavior on a GSN app, without relying on the self-reporting of behavior, is a major strength of our analysis. Similar to previous studies, our results confirm that black men are the most assortative by race with regard to viewing profiles and chatting with candidates. Specifically, our results lend evidence to patterns that suggest that black MSM are highly assortative in their preferences, consistent with propagation of HIV and other STIs on their sexual networks [7,9,29]. Previous studies found that black MSM often had exclusively black partners, had a majority of black partners, or were more likely to select a black partner [4,10]. We found that black searchers were highly assortative in both viewing profiles and chatting across all cities, supporting previous evidence to suggest that black MSM might have less diverse networks than other racial peers [4]. We also found evidence for high assortativity among Asian MSM in Boston, Los Angeles, New York, and San Francisco, suggesting that this may be a group needing targeted intervention. In cities like Tampa and Birmingham, where the overall proportion of black individuals is high, black candidates are being chatted with by all racial groups in these areas.

Viewing someone’s profile is considered a latent interaction and has been found to be extremely common among users of Web-based social networks and is often not reciprocated [30]. Little to no research has been conducted to understand latent interactions on GSN apps targeted at MSM, often because of a lack of available data. A searcher may be more inclined to look at profile details or have a latent interaction with someone in whom they are interested but who may not be perceived as a socially acceptable partner (thus not initiating a chat) [31]. Although this pressure would be expected to matter less in a Web-based setting, because of a lack of observers, these pressures may still exist and should be investigated. It would be presumed that searchers who initiated a chat with a candidate had a reason to do so (ie, desire for interaction, whether on the Web or in person). The fact that more age-disparate chats were initiated suggests a longing for younger partners and friends. Evidence suggests that many of the decisions made in Web-based settings are made purely on the basis of the photograph available on the profile [32]. Although the photograph may indicate phenotypic clues to a candidate’s race and age, we were not able to analyze the content of the photos in relation to the race and age declared of the profile.

### Limitations and Strengths

Caution should be used when employing and interpreting assortativity coefficients as they may mask subgroup behaviors that are important to sexual behavior and STI spread. Specifically, assortativity coefficients are less useful in areas where 1 or 2 groups make up the majority of the population (as in Tampa and Birmingham, in our examples). Therefore, comparing assortativity coefficients across metropolitan areas may only be useful together with information on the distribution of race or age in these areas. In addition, we are only reporting on the behavior of users on a single mobile app. Different mobile apps aimed at connecting MSM have different cultures and composition of users. The observations made from this mobile app may not be generalizable to other groups and apps. An added limitation is that we were not able to examine the behavior of men who did not specify a race or an age in their user profile. These men may have had a reason not to display an age or name (perhaps because they phenotypically belonged to a particular race or age group). The mixing observed is, therefore, limited to those individuals who specified their age or racial group. Finally, assortativity and age preferences are reported here at the population level, potentially obscuring interindividual variability. For example, an individual with 3 to 4 searches (the median) may have different assortativity patterns than an individual with 15,000 searches. These differences in patterns were not explored in this analysis and will be explored in future analyses.

Despite these limitations, this analysis has a number of strengths that provide a unique contribution to the field. Specifically, the results of our analysis include a number of different geographic regions within the United States. Our data also include a large number of MSM of color, and specifically black men, which allows for an objective examination of their behavior on the app. The data we are using are not egocentric or sociometric network data but rather observations on Web-based behavior. Our analysis is, therefore, not likely to be affected by social desirability bias, distortion, self-reporting biases, or recall bias similar to many other analyses examining assortative partnering behaviors among MSM.

### Conclusions

One-size-fits-all interventions aiming to target MSM of color may not work in all contexts or among all minority subgroups. Specific geographic and community-level interventions need to be tailored to complement the needs of each individual population. Although some generalizations can be made regarding Web-based behaviors across all cities, city-specific usage patterns and trends should be analyzed to create targeted and localized interventions that may make the most difference in the lives of MSM in these areas.

## Abbreviations

GPS: global positioning system
GSN: geosocial networking
MSM: men who have sex with men
STI: sexually transmitted infection
